# Regional Expression of *npy* mRNA Paralogs in the Brain of Atlantic Salmon (*Salmo salar*, L.) and Response to Fasting

**DOI:** 10.3389/fphys.2021.720639

**Published:** 2021-08-26

**Authors:** Ingvill Tolås, Tharmini Kalananthan, Ana S. Gomes, Floriana Lai, Sissel Norland, Koji Murashita, Ivar Rønnestad

**Affiliations:** ^1^Department of Biological Sciences, University of Bergen, Bergen, Norway; ^2^Physiological Function Division, Aquaculture Research Department, Fisheries Technology Institute, Japan Fisheries Research and Education Agency, Tamaki, Japan

**Keywords:** Atlantic salmon (*Salmo salar* L.), neuropeptide Y, brain, appetite control, fullness, fasting

## Abstract

Neuropeptide Y (NPY) is known as a potent orexigenic signal in vertebrates, but its role in Atlantic salmon has not yet been fully established. In this study, we identified three *npy* paralogs, named *npya1, npya2, and npyb*, in the Atlantic salmon genome. *In silico* analysis revealed that these genes are well conserved across the vertebrate’s lineage and the mature peptide sequences shared at least 77% of identity with the human homolog. We analyzed mRNA expression of *npy* paralogs in eight brain regions of Atlantic salmon post-smolt, and the effect of 4 days of fasting on the *npy* expression level. Results show that *npya1* was the most abundant paralog, and was predominantly expressed in the telencephalon, followed by the midbrain and olfactory bulb. *npya2* mRNA was highly abundant in hypothalamus and midbrain, while *npyb* was found to be highest expressed in the telencephalon, with low mRNA expression levels detected in all the other brain regions. 4 days of fasting resulted in a significant (*p* < 0.05) decrease of *npya1* mRNA expression in the olfactory bulb, increased *npya2* mRNA expression in the midbrain and decreased *npyb* mRNA expression in the pituitary. In the hypothalamus, the vertebrate appetite center, expression of the *npy* paralogs was not significantly affected by feeding status. However, we observed a trend of increased *npya2* mRNA expression (*p* = 0.099) following 4 days of fasting. Altogether, our findings provide a solid basis for further research on appetite and energy metabolism in Atlantic salmon.

## Introduction

Control of food intake and energy metabolism in vertebrates are complex processes involving several neural pathways. In the brain, the hypothalamus integrates central and peripheral signals that either stimulate (orexigenic) or inhibit (anorexigenic) appetite into a coherent physiological and behavioral response (Volkoff, 2016; Rønnestad et al., 2017; Soengas et al., 2018). Among the signaling molecules involved, neuropeptide Y (NPY) plays a key role. In mammals, it has repeatedly been shown that food deprivation induces increased hypothalamic expression of this neuropeptide, and that administration of the 36 amino acid NPY results in increased food consumption and increased growth and body weight (Reviewed by Beck, 2006; Minor et al., 2009; and Mercer et al., 2011). Concurrently, intake of nutrients lowers the activity of AgRP/NPY neurons, an effect that is proportional to the amounts of calories ingested (Su et al., 2017). In fact, NPY has been reported to be the most potent orexigenic molecule in mammals (Mercer et al., 2011).

Much evidence supports that NPY’s functional role as a regulator of energy homeostasis and appetite control is conserved across vertebrates, including in teleosts (Volkoff et al., 2005; Volkoff, 2016; Rønnestad et al., 2017; Soengas et al., 2018). However, fish are the most diversified group of vertebrates with over 34,000 species identified to date (Froese and Pauly, 2019), and teleosts contain more than half of all vertebrate species (Nelson et al., 2016). This large number of species, along with large variations in anatomy, physiology, habitats and feeding and energy allocation strategies is likely to have caused evolvement of species specific appetite control mechanisms (Volkoff et al., 2009). Indeed, the relative importance of NPY in controlling feed intake seems to vary among teleosts. In several species, including goldfish (*Carassius auratus*) (López-Patiño et al., 1999; Narnaware et al., 2000), grass carp (*Ctenopharyngodon idella*) (Zhou et al., 2013), zebrafish (*Danio rerio*) (Yokobori et al., 2012), and rainbow trout (*Oncorhynchus mykiss*) (Aldegunde and Mancebo, 2006), NPY injections increase feed intake, supporting an orexigenic role. In line with this, food deprivation increased *npy* mRNA expression in the brain of goldfish (Narnaware and Peter, 2001), chinook (*Oncorhynchus tshawytscha*) and coho salmon (*Oncorhynchus kisutch*) (Silverstein et al., 1998), zebrafish (Yokobori et al., 2012) and winter skate (*Leucoraja ocellata*) (MacDonald and Volkoff, 2009b). Concomitantly, refeeding normalized *npy* mRNA abundance following food deprivation in goldfish (Narnaware and Peter, 2001). However, 7 days of fasting did not affect *npy* brain expression in the Atlantic cod (*Gadus morhua*) (Kehoe and Volkoff, 2007), and in cunner (*Tautogolabrus adspersus*) 3 weeks of fasting resulted in a decrease in *npy* expression in the telencephalon (Babichuk and Volkoff, 2013).

In Atlantic salmon (*Salmo salar*), *npy* mRNA expression in the brain did not significantly change after 6 days of fasting (Murashita et al., 2009), but increased during the first 9 h after feeding (Valen et al., 2011). These studies suggests that effects of fasting and feeding in Atlantic salmon central *npy* are time-sensitive and that both spatial and temporal response may be different to that found in mammals. However, the authors analyzed whole brain, an approach that does not take into account regional specific *npy* responses. In fact, NPY has several other functions in the central nervous system besides appetite control, including reproductive regulation (Saha et al., 2015), stress regulation (Reichmann and Holzer, 2016), circadian rhythm (Singh et al., 2017), neurogenesis (Agasse et al., 2008; Baptista et al., 2012), cognition (Redrobe et al., 1999; Gøtzsche and Woldbye, 2016), and visual perception (Santos-Carvalho et al., 2015). Furthermore, due to the four whole genome duplication events (4R WGD) in salmonids, it is expected that Atlantic salmon has several *npy* paralogs with potentially divergent roles. Thus, knowledge about the different paralogs, their regional distribution and their responses to different feeding conditions is key to understand the role of Npy in appetite regulation of Atlantic salmon.

In this study, we provide an *in silico* characterization of the three newly identified Npy paralogs in Atlantic salmon, and investigate the regional brain distribution of *npy* in both fed or 4 days fasted salmon. Additionally, we examine the correlation between gastrointestinal filling and hypothalamic mRNA expression of each *npy* paralog to gain further understanding of the Atlantic salmon gut-brain axis. A 2–4 days fasting period prior to handling, transportation and harvest is common practice in Atlantic salmon aquaculture production (Waagbø et al., 2017), and uncovering impact of fasting on farmed fish is essential to safeguard fish welfare and optimize the aquaculture feeding protocols.

## Materials and Methods

### Ethical Treatment of Animals

The research and sampling were conducted in accordance with the Norwegian Animal Research Authority regulations and was approved by the local representative of Animal Welfare at the Department of Biological Sciences, University of Bergen (Norway).

### Experimental Setup and Sampling

Atlantic salmon post-smolts (ca 180 g) were obtained from Bremnes Seashore’s RAS facility (Trovåg, Norway) and acclimatized in 150 L freshwater tanks at 8.5°C for 18 days. For more details on the experimental setup, please refer to Kalananthan et al. (2020). To evaluate the effect of the fasting, 12 Atlantic salmon post smolts were sampled, 6 from the group that was fed daily *ad libitum* from 9:00 to 16:00 h (sampled 2 h after feeding), and 6 from the 4 days fasted group. The fish were euthanized with an overdose of MS222, and content of the gastrointestinal tract compartments (stomach, midgut, and hindgut) was collected and processed as previously described (Kalananthan et al., 2020). The whole brain was dissected out, and stored in RNAlater (Thermo Fisher Scientific, Waltham, MA, United States). The individual fish weight and length was recorded, and the Fulton’s condition factor (K) determined according to Froese (2006).

### Sequence and Comparative Analysis

The Atlantic salmon *npy* transcripts were searched using the previously identified salmon *npya1* amino acid sequence (GenBank acc. no. NP_001140153.1) as a query against the Atlantic salmon genome database available in NCBI GenBank^1^ and Ensembl^2^. The predicted NPY protein sequences of Atlantic salmon and human NPY were aligned using MUSCLE with the default parameters (UPGMA clustering method, Gap opening penalty −2.90, Gap extension 0.0) from MEGAX (Hall, 2013) and edited using GeneDoc 2.7 software (Nicholas et al., 1997). The percentages of similarity/identity between sequences were calculated using BLASTP^3^. Putative signal peptides were predicted by PrediSi^4^ (Hiller et al., 2004), and mature peptide sequences were predicted using NeuroPred^5^.

Phylogenetic tree was constructed using the deduced amino acid sequences of the full-length NPY from 13 teleost species and the human (*Homo sapiens*) NPY retrieved from NCBI GenBank and Ensembl. Multiple alignments were generated using MUSCLE with the default parameters from MEGAX (Hall, 2013). The sequence alignment was analyzed for the best-fit substitution model in MEGAX to select the best statistical model to study protein family evolution. The phylogenetic tree was constructed using Maximum Likelihood (ML) with a Jones-Taylor-Thornton (JTT) model (Jones et al., 1992) with fixed Gamma distribution (+G) parameter with five rate categories and 1000 bootstrap replicates. The tree was then rooted to the human NPY sequence.

### mRNA Abundance Analysis by RT-qPCR

The Atlantic salmon brain of fed (*n* = 6) and fasted (*n* = 6) fish were dissected into eight regions: olfactory bulb, telencephalon, midbrain, cerebellum, hypothalamus, saccus vasculosus, pituitary, and brain stem. Total RNA was isolated from each brain region using TRI reagent (Sigma-Aldrich) according to the manufacturer’s instructions. Depending on the availability of total RNA per section, 2.5 μg or 10 μg total RNA samples were treated with TURBO DNA-free (Thermo Fisher Scientific) with 1 μl of DNase (2 U/μl) in 10 or 30 μl reaction volume, respectively, to eliminate possible genomic DNA contamination. Quantity and integrity of DNase treated total RNA was assessed using a NanoDrop ND-1000 spectrophotometer (Thermo Fisher Scientific, Waltham, MA, United States) and an Agilent 2100 Bioanalyzer (Agilent Technologies, Santa Clara, CA, United States). cDNA was synthesized from 1.1 μg of DNase treated total RNA using oligo (dT) primer from SuperScript III First-Strand Synthesis system for RT-PCR kit (Thermo Fisher Scientific). Specific primers spanning an exon-exon junction were designed for all the target genes (Supplementary Table 1). β-*actin* and *ribosomal protein s20* (*s20*) were used as reference genes. qPCR reactions were performed in duplicate using iTaq Universal SYBR Green Supermix (Bio-Rad, Hercules, CA, United States) in a 20 μl final reaction volume. The qPCR reactions were performed in a Bio-Rad CFX96^TM^ Real-Time System with the following cycling conditions: 95°C for 30 s; 40 cycles of 95°C for 5 s, 60°C for 25 s. Melting curve analysis over a range of 65–95°C (increment of 0.5°C for 2 s) allowed for the detection of possible nonspecific products and/or primer dimers.

Standard curves were generated by the protocol described by Kalananthan et al. (2020) and used to determine the qPCR efficiency for each assay (Supplementary Table 1).

### Statistical Analysis

Statistical analyses were performed using GraphPad (GraphPad Software, version 9). Equality of variances and normality of data related to fish weight, length, K, gastrointestinal content, and gene expression were tested using *F*-test and Shapiro–Wilk normality test, respectively. Grubb’s outlier test was used prior to statistical evaluations and outliers were removed. Analysis of differential expression between the fed and fasted group within a brain region and differences between the fed and fasted group pertaining to K and gastrointestinal filling was performed with two-tailed *t*-test. When either the *F*-test or the normality test failed, the no-parametric Mann–Whitney test was performed. Pearson’s correlation coefficients were calculated to investigate the correlation between wet and dry weight content of gastrointestinal compartments (stomach, midgut, and hindgut), as well as between hypothalamic *npy* mRNA expression and dry weight content from each section of the gastrointestinal tract normalized by fish weight. A *p* < 0.05 was considered significant. All data are presented as mean ± SEM, unless otherwise stated.

## Results

### Characterization of Atlantic Salmon Npy and Phylogenetic Analysis

**FIGURE 1:**
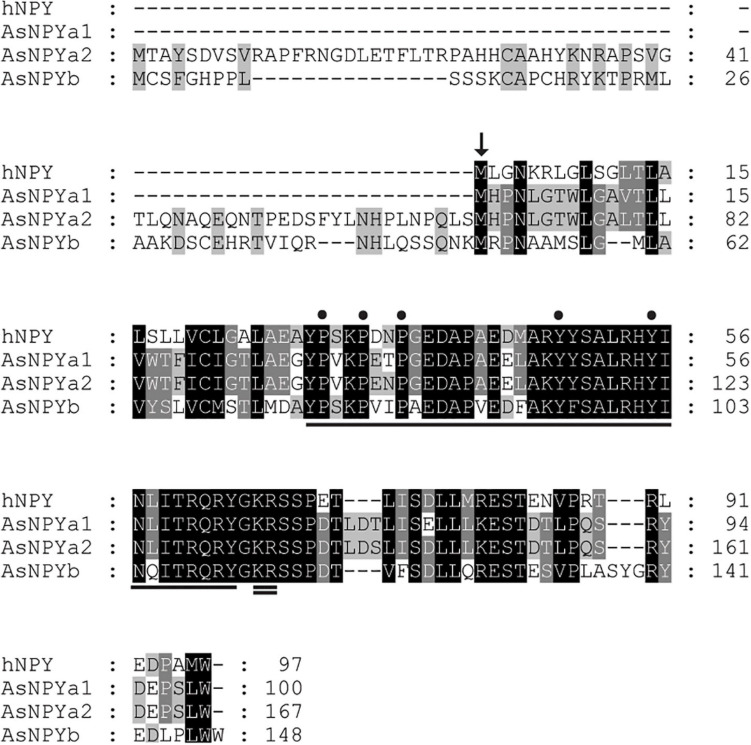
Primary protein sequence alignment of the human neuropeptide Y (NPY) and the Atlantic salmon NPY paralogs (NPYa1, a2 and b). Arrow indicates the beginning of the predicted signaling peptide for the human NPY. Underline indicates the mature NPY sequence and double underline indicates the conserved processing amino acid sites (KR). The three proline (P) and two tyrosine (Y) residues, which are imperative to the conformation of the NPY family, are indicated by dots.

In Atlantic salmon, three *npy* genes were found to be located on chromosomes ssa14 (*npya1*), ssa27 (*npya2*), and ssa5 (*npyb*). The predicted full length amino acid (AA) sequences of Atlantic salmon NPY varied from 100 to 167 AA in length (data retrieved from Ensembl, October 2020) (Supplementary Figure 1), with predicted protein masses between 11.33 and 18.83 kDa (predicted by Expasy)^6^ (Supplementary Table 2). The predicted pro-NPY peptides contained putative signal peptides of 28, 95, and 75 AAs for the NPYa1, NPYa2, and NPYb paralogs, respectively [PrediSi (see text footnote 4), Hiller et al., 2004; Figure 1]. The potential processing signal (KR) at the C-terminal of the mature peptide was found to be well conserved (Figure 1). The predicted 36 AA mature sequences showed a molecular weight around 4.2–4.3 kDa (Supplementary Table 2). Based on the predicted mature peptide sequences, NPYa1 and NPYa2 shared 97% identity at the AA level, and both shared 75% identity with NPYb. All three paralogs are relatively well conserved with the human homolog, sharing between 78 and 86% AA sequence identity, with NPYa2 being most similar (Supplementary Table 3). The three proline and two tyrosine residues vital to the conformation of the NPY family were conserved in all three salmon NPY paralogs (Figure 1; Cerda-Reverter and Larhammar, 2000).

**FIGURE 2:**
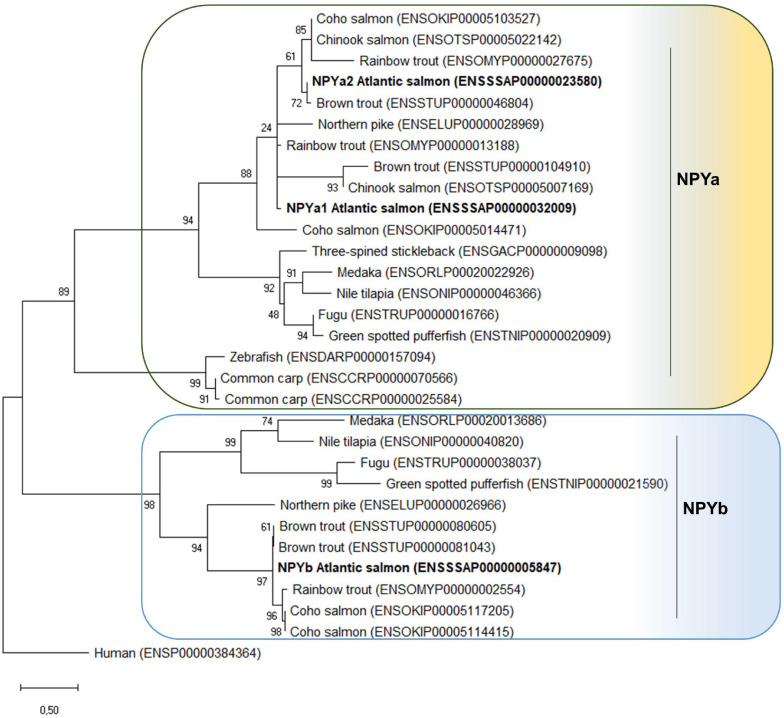
Phylogenetic tree of the NPY family peptide in teleosts. The phylogenetic tree was constructed based on the NPY predicted full-length amino acid sequences using the Maximum Likelihood method and JTT matrix-based model with fixed Gamma distribution parameter with five rate categories, and 1000 bootstrap replicates. The tree was rooted to the human NPY sequence. Evolutionary analyses were conducted in MEGAX (Hall, 2013). Accession numbers are provided in front of the common species name.

Phylogenetic analysis showed that the NPY peptides encoded by the three Atlantic salmon *npy* genes group with teleost homologs (Figure 2). Two major clades are present, one containing the teleost NPYa and the other NPYb. Most teleost species have one *npya* and one *npyb* gene, however, Cypriniformes only have one *npya* (zebrafish) or two *npya* genes (common carp (*Cyprinus carpio*)). In the case of salmonids, all species analyzed have 2 *npya* genes. However, for NPYb, the case is different: two *npyb* genes were found for coho salmon and brown trout (*Salmo trutta*), while only one *npyb* gene was present for Atlantic salmon and rainbow trout and no *npyb* gene was found in the Chinook salmon genome.

### Brain Distribution of Atlantic Salmon *npy* mRNA

**FIGURE 3:**
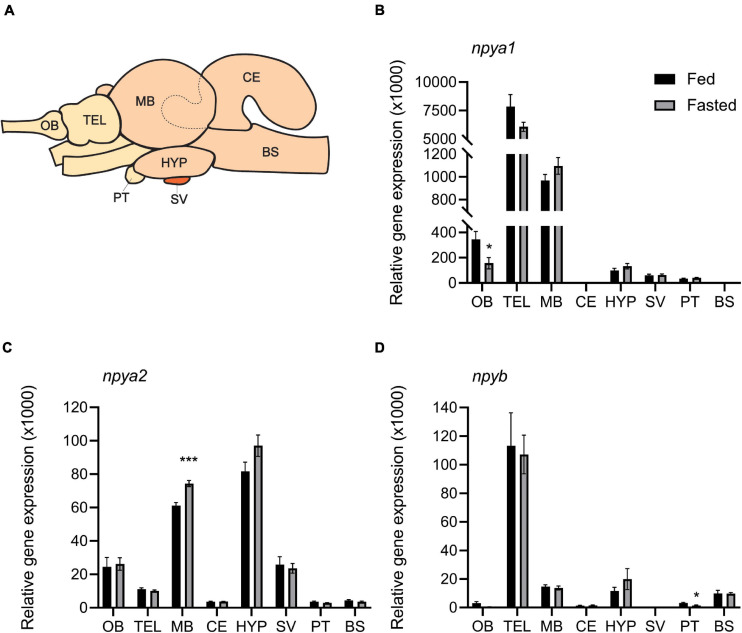
**(A)** Schematic representation of Atlantic salmon brain, showing dissection of the eight brain regions used for gene expression analysis: olfactory bulb (OB), telencephalon (TEL), midbrain (MB), hypothalamus (HYP), cerebellum (CE), saccus vasculosus (SV), pituitary (PT), and brain stem (BS). The dashed line represents the dissected area of the CE inside the MB. Effects of 4 days of fasting on the mRNA expression levels of **(B)** *npya1*, **(C)** *npya2*, and **(D)** *npyb* in the eight regions of Atlantic salmon brain. Black and gray columns represent fed and fasted fish, respectively. *n* = 6 for both groups and for all regions except in saccus vasculosus (*n* = 4) and in instances where technical or statistical outliers have been removed (refer to Supplementary Table 5). Values are expressed as copy number per ng total RNA used in the reaction normalized using the geometric mean of the reference genes s20 and β-actin-actin. Bars represent mean ± SEM. Two-tailed *t*-test was performed to assess whether there were statistically significant differences between the fed and fasted group for each brain region. Asterisks (*) indicate statistically significant difference (*p ≤ 0.05, ***p ≤ 0.001).

The three *npy* paralogs showed a wide distribution in the eight brain regions analyzed (Figure 3). *npya1* mRNA was found to be highly expressed in the telencephalon, followed by the midbrain and olfactory bulb. *npya2* mRNA level were highly abundant in the hypothalamus, midbrain, olfactory bulb, saccus vasculosus, and telencephalon, while mRNA expression levels of *npyb* was found to be highest in the telencephalon, with lower levels of expression in the hypothalamus, midbrain and brain stem, and only residual mRNA expression levels in the other brain regions. Overall, the *npya1* was the most abundant paralog in the Atlantic salmon brain, and all *npy* paralogs showed very low mRNA expression levels in the cerebellum, pituitary, and brain stem. Although very low, *npy* detected in the cerebellum is noteworthy as expression found in this region is rare.

### Effects of 4 Days of Fasting on Atlantic Salmon *npy*

**FIGURE 4:**
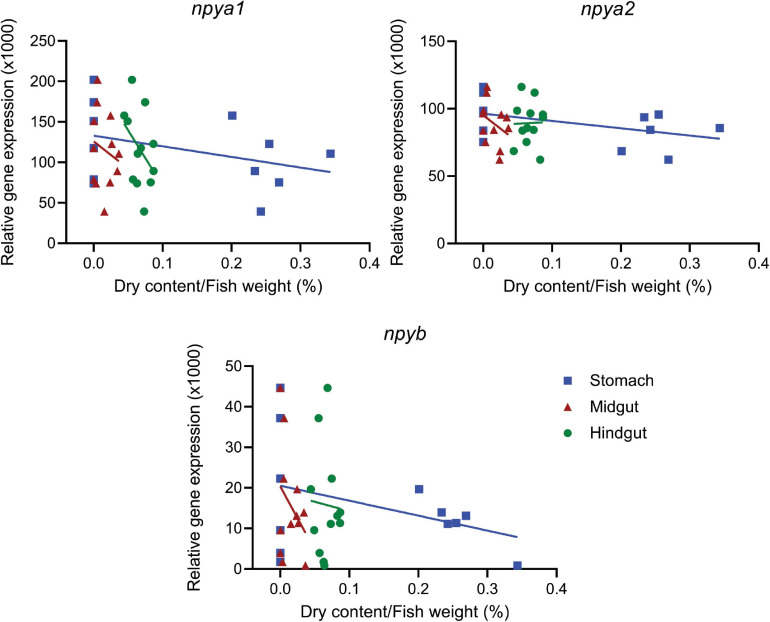
Correlation between dry content in the gastrointestinal tract and hypothalamic expression of *npya1*, *npya*2, and *npyb*. The plots show hypothalamic mRNA expression of *npya1*, *npya2* and *npyb versus* content in the stomach, midgut and hindgut. Dots represent individual fish (*n* = 12), while solid lines represent the linear regression. The dry weight of the gastrointestinal content was standardized to the fish weight. For detailed statistical information, refer to Supplementary Table 6.

Both fed and fasted fish groups had a mean *K* factor of 1.11 (Supplementary Figure 2A). As expected, there was a significant (*p* < 0.0001) correlation between wet and dry digesta weight in all gastrointestinal tract compartments (Supplementary Figure 2B and Supplementary Table 4). The amount of digesta in the stomach and midgut of fasted fish was, as expected, significantly (*p* < 0.05) lower compared to the fed group. However, there were no differences between the two groups in the hindgut (Supplementary Figure 2C and Supplementary Table 4). Four days of fasting resulted in significantly (*p* < 0.05) decreased expression of *npya1* in the olfactory bulb and *npyb* in pituitary, and increased *npya2* expression in the midbrain. In addition, we also observed a trend (*p* = 0.99) of increased hypothalamic expression of *npya2* in fasted fish compared to fed (Figure 3 and Supplementary Table 5). No statistically significant correlation was found between hypothalamic mRNA expression of *npy* paralogs and the inner content of the gastrointestinal compartments. The highest observed correlation was between stomach content and *npya2* mRNA expression (*p* = 0.129) (Figure 4 and Supplementary Table 6).

## Discussion

In the present study, we report for the first time the identification and characterization of three Atlantic salmon *npy* paralogs; namely *npya1*, *npya2*, and *npyb.* All paralogs were highly conserved at the amino acid level, sharing between 78 and 86% sequence identity with the human homolog. Moreover, in agreement with previous findings (Murashita et al., 2009), each paralog encoded the three proline and two tyrosine residues (Pro^2/5/8^ and Tyr^20/27^) known to be important for maintaining protein conformation (Figure 1; Cerda-Reverter and Larhammar, 2000). In teleosts, the NPY peptides so far described have been named NPYa and NPYb (Figure 2). Presumably, the a and b duplicates have resulted from the WGD in the teleost fish lineage (Sundström et al., 2008). Differential losses may have occurred, since zebrafish, common carp, three-spined stickleback (*Gasterosteus aculeatus*) and Chinook salmon seems to be missing NPYb. Equally, the additional *npya* gene duplication is present for both salmonids and common carp, as most probably a result of the additional 4R WGD duplication in salmonids and carps (Tang et al., 2014). This was, however, not observed for *npyb* gene, suggesting that *npyb* gene duplication or absence may be a result of a species-specific event. Importantly, this is the first report of NPYb being present in the Atlantic salmon genome and it is likely owed to the recently updated databases.

The predicted NPY protein sequence conservation likely points toward a conserved functional role NPY as a regulator of energy metabolism in Atlantic salmon. However, conclusions from previous studies have been somewhat divergent, possibly due to a temporal response of *npy* expression to feeding status. The results from Murashita et al. (2009) indicated that 6 days fasting did not increase expression levels of *npy*, while Valen et al. (2011) found that feeding status significantly affected *npy* expression levels in the brain, with the increase taking place during the first 9 h after feeding. Importantly, the primers used in these studies only amplified *npya*, and most likely did not differentiate between *npya1* and *npya2.* Moreover, both studies used the whole brain for gene expression analysis, an approach which does not consider potential region-specific responses in *npy* expression or possibly distinct expression pattern of each paralog gene. This is especially important given that the presence of multiple paralogs opens the possibility for neofunctionalization and/or subfunctionalization (Lien et al., 2016).

To overcome this limitation, we investigated the expression pattern of each *npy* gene, and their response to 4 days of fasting in eight brain regions. *npya1* was found to be the most abundant paralog and was highly expressed in the telencephalon, followed by the midbrain, olfactory bulb and hypothalamus (Figure 3B). Interestingly, expression in the olfactory bulb decreased significantly following 4 days of fasting. Previous studies have identified the presence of NPY in the olfactory bulb in teleost species such as goldfish (Pontet et al., 1989), the ayu (*Plecoglossus altivelis*) (Chiba et al., 1996) and zebrafish (Kaniganti et al., 2019). In zebrafish it was also demonstrated that NPY signaling increased upon fasting, indicating that NPY levels in the olfactory bulb reflect the energy status in the brain (Kaniganti et al., 2019). Though we observed the opposite effect, our findings may indicate that *npya1* plays a role in the olfactory input related to energy regulation in Atlantic salmon. Moreover, its increased expression in this brain region may account for observations made by Valen et al. (2011). However, we cannot rule out that expression in other brain regions, such as the hypothalamus, may have been affected in the immediate or short-term time frame used in that study. The high expression in the telencephalon is also of interest to the field of appetite control since this brain region has been proposed to be the location of mechanisms involved in hedonic regulation of food intake in fish (O’Connell and Hofmann, 2011; Comesaña et al., 2018; Otero-Rodiño et al., 2018).

*npya2* was found to be highest expressed in the hypothalamus and while its expression did not significantly change depending on feeding status, there was a trend of increased expression following 4 days of fasting (*p* = 0.99) (Figure 3C). The hypothalamus is considered the hub for appetite control in vertebrates, and in spite of not being statistically significant, this finding may support the suggested role of NPY as an orexigenic factor in vertebrates, including several fish species, such as goldfish (López-Patiño et al., 1999; Narnaware and Peter, 2001), grass carp (Zhou et al., 2013), zebrafish (Yokobori et al., 2012; Tian et al., 2015), rainbow trout (Aldegunde and Mancebo, 2006), winter skate (MacDonald and Volkoff, 2009b), Atlantic cod (Kehoe and Volkoff, 2007), tiger puffer (*Takifugu rubripes*) (Kamijo et al., 2011), nile tilapia (*Oreochromis niloticus*) (Yan et al., 2017), chinook and coho salmon (Silverstein et al., 1998).

Notably, expression of both *npya1* and *npya2* was found to be high in midbrain, and expression of *npya2* mRNA increased significantly following 4 days fasting (Figures 3B,C). The implications of this depends on the area of the midbrain affected. *npy* mRNA has been observed in the *optic tectum* region of midbrain in zebrafish (Söderberg et al., 2000), goldfish (Peng et al., 1994; Narnaware and Peter, 2001), Coho and Chinook salmon (Silverstein et al., 1998), Atlantic cod larva (Le et al., 2016), sea bass (Cerdá-Reverter et al., 2000), winter flounder (*Pseudopleuronectes americanus*) (MacDonald and Volkoff, 2009a) and winter skate (MacDonald and Volkoff, 2009b). Moreover, NPY-immunoreactive structures have been identified in the *optic tectum* of Atlantic salmon, as well as several other fish species (Vecino and Ekström, 1990; Chiba, 2005; Pirone et al., 2008; Amiya et al., 2011; Magliozzi et al., 2019). The *optic tectum* is the visual center in the non-mammalian brain, and while the function of NPY in this brain region of fish remains to be fully elucidated, the presence of *npya1* and *a2* could indicate a role in modulating retinotectal relay, as proposed in domestic chicks (*Gallus gallus domesticus*) (Székeley et al., 1992) and in toad *Bombina orientalis* (Funke and Ewert, 2006). If the observed increased *npya2* expression occurs in this region, it would be in line with a previous study in goldfish (Narnaware and Peter, 2001) and could indicate a link between feeding status and visual perception in Atlantic salmon. Such a link has previously been demonstrated in zebrafish (Filosa et al., 2016), and may be part of a broader physiological response in which hunger triggers a shift from escape to approach in the case of limited food availability (Barker and Baier, 2015). Another important region of the midbrain is the preoptic area (POA), which is involved in thermoregulation, mating behavior and orexin detection (Nelson and Prosser, 1979, 1981; Kaslin et al., 2004; Yokobori et al., 2011; Tripp et al., 2020) and is a site for Npy-action in several fish species including goldfish (Narnaware and Peter, 2001), Senegalese sole (*Solea senegalensis*) (Rodríguez-Gómez et al., 2001), pejerrey (*Odontesthes bonariensis*) (Traverso et al., 2003), Cichlid fish (*Cichlasoma dimerus*) (Pérez Sirkin et al., 2013), African lungfish (*Protopterus annectens*) (Trabucchi et al., 2000) and Coho and Chinook salmon (Silverstein et al., 1998). However, expression and possible function of *npya1* and *a2* in the POA of Atlantic salmon remains to be elucidated. If increased expression of *npya2* occurs in this area of the midbrain, it would be in line with findings in goldfish (Narnaware and Peter, 2001) and Chinook and Coho salmon (Silverstein et al., 1998) that the POA is key in orexin detection, thermoregulation and mating behavior. Since these are all tightly intertwined with energy homeostasis and appetite (Peyon et al., 1999; Volkoff and Rønnestad, 2020), a link between feeding status and *npy* expression level in this region would be unsurprising. While these are alluring speculations, tailored studies will be required to assess the plausibility.

For *npyb* (Figure 3D) fasting only affected mRNA expression levels in the pituitary. Given the very low expression of *npyb* in the pituitary and that there is no known correlation between pituitary and appetite regulation, this is likely an artifact. In sum, our findings therefore suggest that Atlantic salmon *npyb* is likely not involved in central appetite control. This is in agreement with previous studies in Nile Tilapia, where it was found that *npya* is the main paralog involved in feeding regulation (Yan et al., 2017), as well as in tiger puffer, where it was found that *npya*, and not *npyb*, in the hypothalamus is involved in regulating feed intake (Kamijo et al., 2011). Previous studies in fish have indicated that Npy has a widespread distribution, with expression found in the brain, gastrointestinal tract (summarized by Volkoff, 2016) and eye (Kurokawa and Suzuki, 2002; Chen et al., 2005; Murashita et al., 2009). As such, the NPYb might serve its main function in one of these tissues. We also cannot rule out NPYa1 and NPYa2 having different functions in unexamined tissues, and a study exploring *npy* expression in peripheral tissues would be of high interest.

Another important aspect to consider when studying central appetite control is its relationship to peripheral signaling. Distention of the stomach and interactions between nutrients and receptors on the gut wall regulates secretion of peptide hormones that communicate the degree of stomach and gut fullness as well as nutritional content to the central system. The gut-brain axis is key in control of food intake during a meal (Grove et al., 1978; Sam et al., 2012; Kalananthan et al., 2020), and we therefore investigated the relationship between hypothalamic expression of the *npy* paralogs and content of the gastrointestinal compartments. While no statistically significant correlation was found, the highest observed correlation was that between stomach content and hypothalamic *npya2* expression (Figure 4 and Supplementary Table 6). Given the inverse relationship between appetite and fullness (Grove et al., 1978; Sam et al., 2012; Kalananthan et al., 2020), this could support the hypothesis that *npya2* encodes an important orexigenic factor in Atlantic salmon.

In summary, we identified three *npy* paralogs in the Atlantic salmon genome, including *npyb*, and demonstrated a significant effect of fasting on expression of *npya1* in the olfactory bulb, *npya2* in the midbrain and a trend toward increased expression of *npya2* in the hypothalamus. These findings support a conserved role of NPY in appetite control.

## Data Availability Statement

The original contributions presented in the study are included in the article/Supplementary Material, further inquiries can be directed to the corresponding author/s.

## Ethics Statement

The experiment was conducted in an experimental facility approved to conduct experiments with teleosts and in accordance with the rules and regulations of the Norwegian Animal Research authority. The experiment was approved by the local representatives for animal welfare at the Department of Biological Sciences, University of Bergen (Norway). The personnel conducting the experiment and sampling were accredited by Federation of European Laboratory Animal Science Associations (FELASA).

## Author Contributions

IT, TK, FL, KM, IR, and AG designed the study. TK, FL, and AG did the preparatory lab work. IT performed the qPCR analysis. IT and FL did the statistical analysis. AG did the phylogeny analysis. IR made the basis for the schematic illustration of the Atlantic salmon brain and oversaw the project. All authors contributed to data analysis and writing of the manuscript, and approved the final version.

## Conflict of Interest

The authors declare that the research was conducted in the absence of any commercial or financial relationships that could be construed as a potential conflict of interest.

## Publisher’s Note

All claims expressed in this article are solely those of the authors and do not necessarily represent those of their affiliated organizations, or those of the publisher, the editors and the reviewers. Any product that may be evaluated in this article, or claim that may be made by its manufacturer, is not guaranteed or endorsed by the publisher.

## Footnotes

1
https://www.ncbi.nlm.nih.gov/genbank/

2
http://www.ensembl.org/index.html

3
https://blast.ncbi.nlm.nih.gov/Blast.cgi

4
http://www.predisi.de/home.html

5
http://stagbeetle.animal.uiuc.edu/cgi-bin/neuropred.py

6
https://www.expasy.org/
