# Supplementary material for: Regional Expression of npy mRNA Paralogs in the Brain of Atlantic Salmon (Salmo salar, L.) and Response to Fasting
Source: Front Physiol. 2021 Aug 26;12:720639. doi: 10.3389/fphys.2021.720639 (PMC8427667; doi:10.3389/fphys.2021.720639)
Supplement: Supplementary file 1 [file Data_Sheet_1.PDF]

Supplementary Material

Supplementary Figures

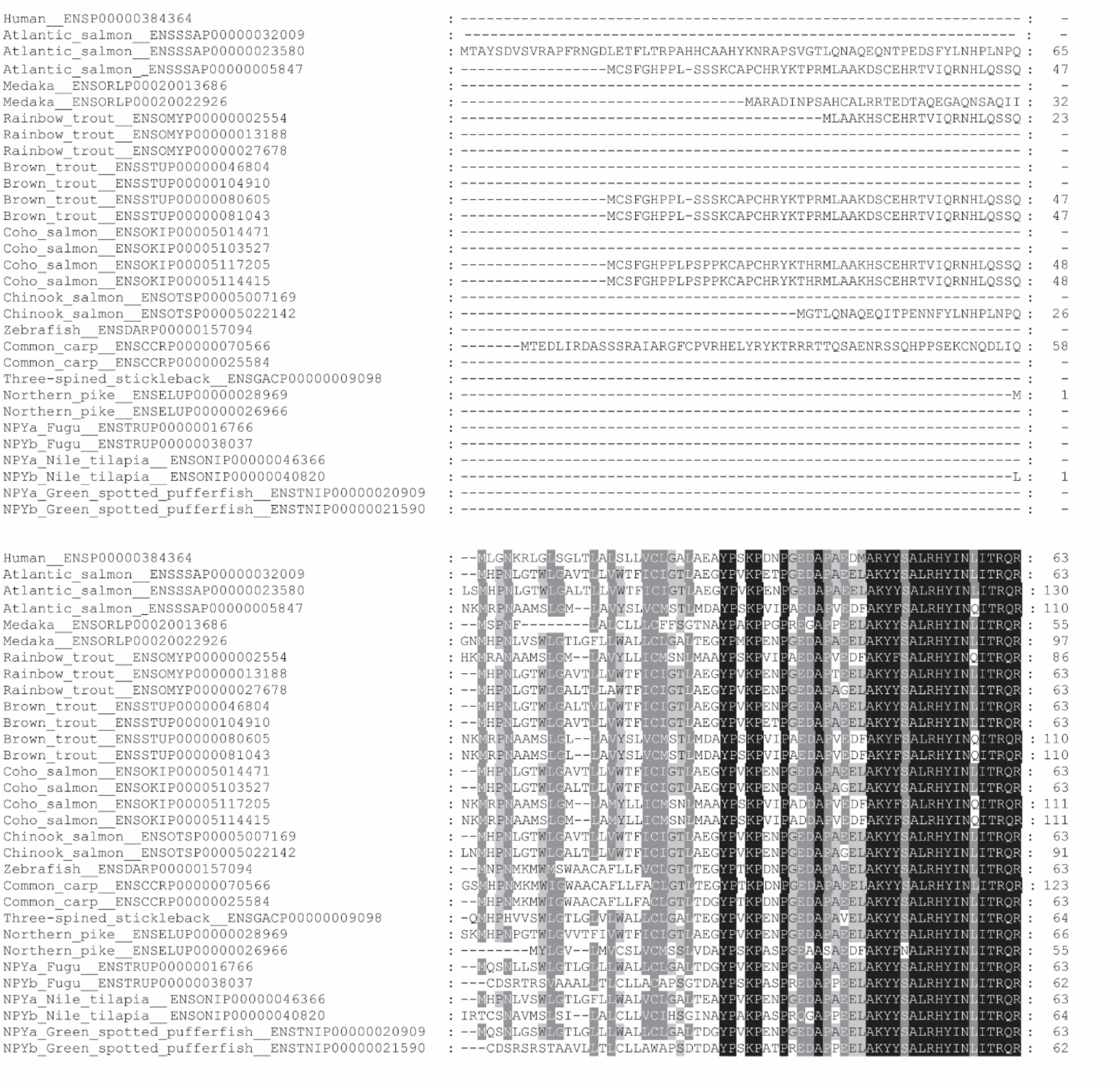

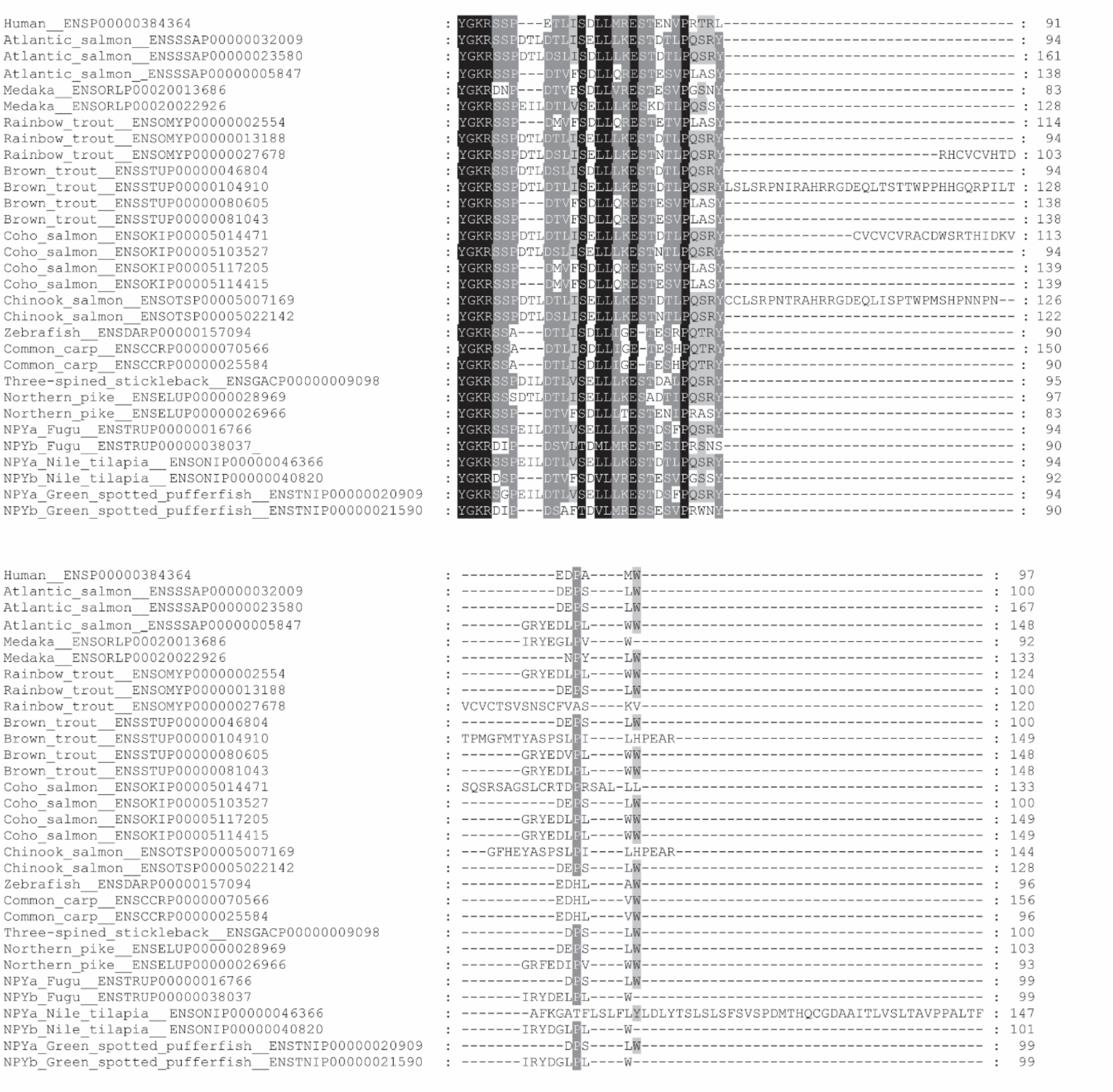

Supplementary Figure 1. Multiple sequence alignment of NPY. The predicted amino acid sequences of the Atlantic salmon NPY were aligned with those of other teleost species using Multiple sequence alignment in MUSCLE. Conserved amino acids among the species are highlighted in black.

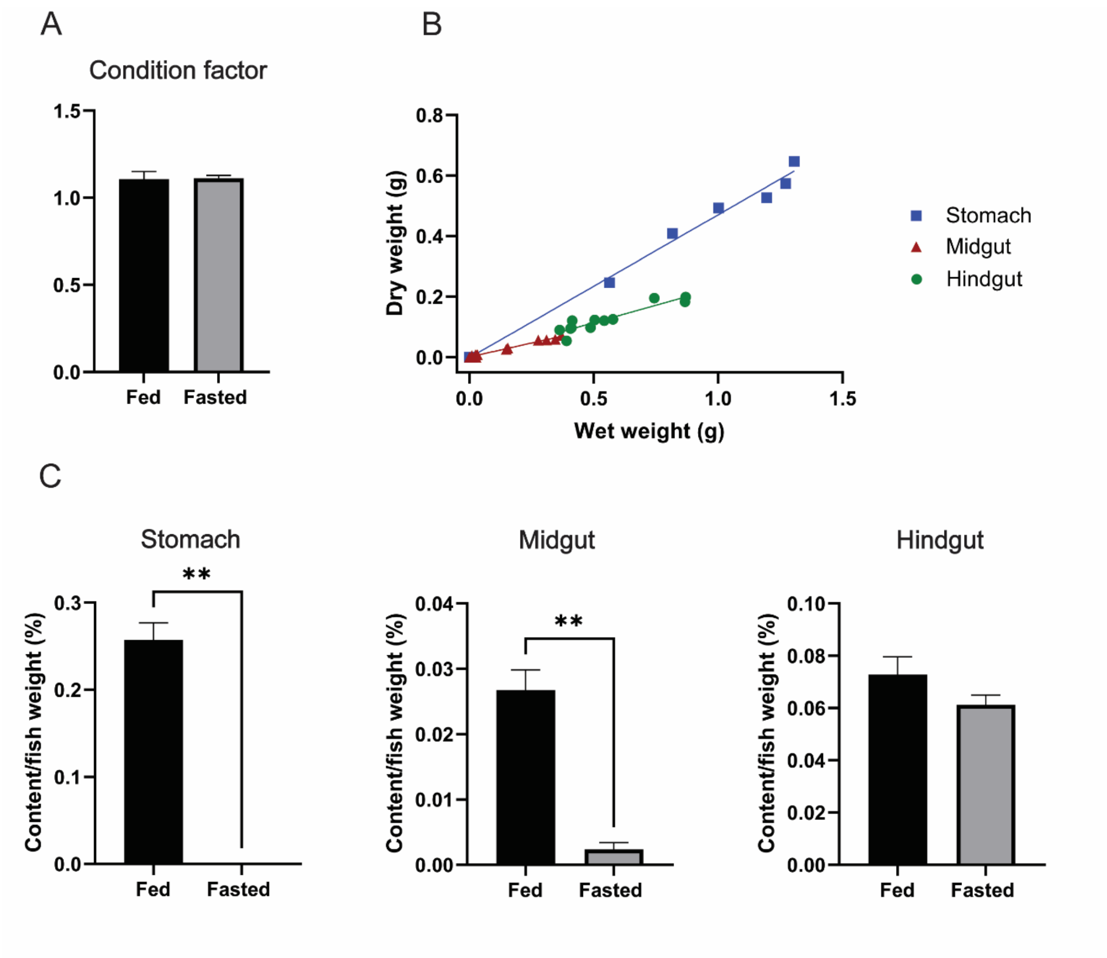

Supplementary Figure 2 – A) Condition factor (K) in fed and fasted Atlantic salmon. **B)** Correlation between dry weight (g) and wet weight (g) content for stomach, midgut and hindgut. Dots represent individual fish (n = 12), while solid lines represent the linear regressions. p-values following Pearson's correlation analysis were < 0.0001**** for all regions. **C)** Stomach, midgut and hindgut dry content standardized by the fish weight. Results are presented as mean ± SEM (*n* = 6) and asterisks indicate statistically significant differences; **p < 0.01. For detailed statistical information, refer to Supplementary table 4.

Supplementary tables

Supplementary table 1 - Sequence of the specific primers used for qPCR mRNA expression analysis. Primer sequences, amplicon sizes, R^2, and qPCR efficiency are indicated for each primer pair

Gene	Ensembl gene acc.no.	Sequence 5' → 3'	Amplicon length (bp)	R^2	Efficiency (%)
npya1	ENSSSAG00000040508	F:GAACGCACAGCAGCAGAAAG<br>R:AGGATGCATATTGACTTGAAGGTT	80	0.9979	110
npya2	ENSSSAG00000015791	F:CAGTCCAGGTATGATGAACCGT<br>R:GGCACAGGAGTAACCTCTGG	195	0.9974	103
npyb	ENSSSAG00000002935	F:CCAAACCTGTCATTCCCGCA<br>R:CTTTTCCCATACCGCTGTCG	109	0.9982	114
β-actin	ENSSSAG00000001782	F: CCAAAGCCAACAGGGAGAAG<br>R: AGGGACAACACTGCCTGGAT	91	0.9995	97
s20	ENSSSAG00000073156	F: GCAGACCTTATCCGTGGAGCTA<br>R: TGGTGATGCGCAGAGTCTTG	85	0.9975	100

Supplementary Table 2: Databank accession number in Ensembl[1] and Genbank[2], chromosome placement, predicted gene length, predicted amino acid (AA) sequence for full length, without signaling peptide and mature peptide and predicted masses for each of the Atlantic salmon NPYs.

Gene	Protein	Ensembl acc.no	GenBank acc.no	Length (bp)	Chr	Ensembl protein acc.no	Full length (AA)	Predicted mass (kDa)	Without signaling peptide (AA)	Predicted mass without signaling peptide (kDa)	Mature peptide (AA)	Predicted mass mature peptide (kDa)
npya1	AsNPYa1	ENSSSAG00000040508	NM_001146681.1	328	ssa14	ENSSSAP00000032009	100	11.,33	72	8.33	36	4.25
npya2	AsNPYa2	ENSSSAG00000015791	XM_014178359.1	1513	ssa27	ENSSSAP00000023580	167	18.83	72	8.31	36	4.27
				1585	ssa27	ENSSSAP00000023586	191					
npyb	AsNPYb	ENSSSAG00000002935	XM_014202299.1	789	ssa05	ENSSSAP00000005847	148	16.87	73	8.55	36	4.28

Supplementary Table 3: Percent identity between Atlantic salmon NPY paralogs and human NPY for the mature peptide

	**AsNPYa1**	**AsNPYa2**	**AsNPYb**
**AsNPYa2**	97.22 %		
**AsNPYb**	75.00 %	75.00 %	
**hNPY**	83.33 %	86.11 %	77.78 %

[1] https://www.ensembl.org/index.html

[2] https://www.ncbi.nlm.nih.gov/genbank/

Supplementary Table 4: Statistical analysis of information pertaining to fish and gastrointestinal content.

	Average fed	Average fasted	Shapiro Wilk	F-test	Test	p-value
**Condition factor**	1.11 ± 0.04	1.11 ± 0.02	Passed	Passed	t-test	0.9251
**Weight (g)**	188.9 ± 18.98	178.7 ± 10.10	Passed	Passed	t-test	0.6478
**Length (cm)**	25.62 ± 0.82	25.22 ± 0.59	Passed	Passed	t-test	0.6998
**Stomach (dry content/fish weight (%))**	0.2573 ± 0.0196	0 ± 0.0000	Invalid for fasted	Failed	Mann-Whitney	**0.0022****
**Midgut (dry content/fish weight (%))**	0.0267 ± 0.0031	0.0024 ± 0.0010	Passed	Failed	Mann-Whitney	**0.0022****
**Hindgut (dry content/fish weight (%))**	0.0728 ± 0.0068	0.0612 ± 0.0038	Passed	Passed	t-test	0.1652

Supplementary Table 5: Statistical test and p-values for each gene expression per brain region between treatments

Brain region	*npya1*				*npya2*				*npyb*			
	Shapiro-Wilk	n fed;fasted	Test	p-value	Shapiro-Wilk	n fed;fasted	Test	p-value	Shapiro-Wilk	n fed/fasted	Test	p-value
**OB**	passed	6;5	t-test	**0.044***	passed	6;6	t-test	0.809	passed	6;6	Mann-Whitney	0.065
**TEL**	passed	6;6	t-test	0.154	passed	5**;6	t-test	0.281	passed	6;6	t-test	0.825
**MB**	passed	6;6	t-test	0.187	passed	5**;6	t-test	**0.0007*****	passed	6;6	t-test	0.597
**CE**	passed	6;6	t-test	0.237	passed	6;6	t-test	0.784	passed	6;6	t-test	0.639
**HYP**	passed	6;6	t-test	0.240	passed	6;6	t-test	0.099	passed	6;6	Mann-Whitney	0.699
**SV**	passed	4;4	t-test	0.899	passed	4;4	t-test	0.712	passed	4;4	t-test	0.296
**PT**	passed	6;6	t-test	0.316	passed	5**;6	Mann-Whitney	0.178	passed	5**;6	t-test	**0.019***
**BS**	failed	6;6	Mann-Whitney	>0.999	passed	6;6	t-test	0.246	passed	6;6	Mann-Whitney	0.818

**Statistically significant **Outlier removed following Grubb's outlier test*

Supplementary Table 6: Results from the Pearson correlation analysis for the hypothalamic *npy* mRNA expression levels *versus* gastrointestinal tract dry weight content normalized to fish weight (n=12). Correlation coefficient, 95% confidence interval and p-value are given.

Gene	**Gut compartment**	**R^2**	**r (95% confidence interval)**	**p-value**
npya1	Stomach	0.1432	-0.3785 (-0.7824; 0.2497)	0.225
	Midgut	0.0375	-0.1937 (-0.6908; 0.4277)	0.546
	Hindgut	0.1666	-0.4081 (-0.7957; 0.2165)	0.188
npya2	Stomach	0.2154	-0.4641 (-0.8197; 0.1496)	0.129
	Midgut	0.1163	-0.3411 (-0.7652; 0.2895)	0.278
	Hindgut	0.0006	0.0247 (-0.5571; 0.5902)	0.939
npyb	Stomach	0.1429	-0.3781 (-0.7822; 0.2501)	0.226
	Midgut	0.1037	-0.3221 (-0.7562; 0.3089)	0.307
	Hindgut	0.0024	-0.0490 (-0.6058; 0.5401)	0.880
